# Data Driven Approach to the Dynamics of Import and Export of G7 Countries

**DOI:** 10.3390/e20100735

**Published:** 2018-09-25

**Authors:** Gianluca Teza, Michele Caraglio, Attilio L. Stella

**Affiliations:** 1 Dipartimento di Fisica e Astronomia Università di Padova, Via Marzolo 8, I-35131 Padova, Italy; 2 KU Leuven, Institute for Theoretical Physics, Celestijnenlaan 200D, 3001 Leuven, Belgium; 3 Sezione INFN, Università di Padova, Via Marzolo 8, I-35131 Padova, Italy

**Keywords:** countries-products network, export dynamics, economic complexity

## Abstract

The dynamics of imports plus exports of 226 product classes by the G7 countries between 1962 and 2000 is described in terms of stochastic differential equations. The model allows interesting comparisons among the different economies related to the compositions of the national baskets. Synthetic solutions can also be used to estimate hidden and unexploited growth potentials. These prerogatives are strictly connected with the fact that a network structure is at the basis of the model. Such a network expresses the mutual influences of different products through resource transfers, and is a key ingredient producing cooperative growth effects which can be quantified and distinguished from those generated by deterministic drifts and representing direct resource inputs. An analysis of this network, which differs substantially from those previously considered within the economic complexity approach, allows to estimate the centrality of different products in each national basket, highlighting the most essential commodities for each economy. Solutions of the model give the possibility of performing counterfactual analyses aimed at estimating how much the growth of each country could have profited from a general strengthening, or weakening, of the links in the same products network.

## 1. Introduction

Recently, the availability of extensive data-sets concerning a large number of product groups exported and imported by all nations stimulated investigations aimed at determining diversities or other complexity features of economic systems. In particular, the approaches to economic complexity [1,2] or Fitness [3,4,5,6] try to extract from the panel of yearly export data information on the degrees of diversification of national economies consistently with the specialization of the products. In all these approaches, even if dynamic considerations are the final objective, the data contained in the bipartite network connecting, e.g., countries to exported products, are used to construct indicators applying year by year. Only in a later stage these indicators are used in combination with other quantities, like gross domestic product per capita (GDP_pc_), in regression or alternative analyses of dynamic character [6].

A different approach consists in considering the yearly data as a particular realization of a stochastic model process, which, once identified and calibrated, should give more direct insight into dynamics. The idea to model the time evolution of country-product networks by such processes has been put forward most recently and shown to open the possibility of insight into complexity features directly related to dynamics and not otherwise accessible [7,8]. A first step in this direction was the formulation of a model for the dynamics of exports globally aggregated among all countries in the world [7]. This model can be considered as a reference one for treating the dynamics of exports, or exports plus imports, in a specific country for several reasons [8]. In first place, there is a clear tendency, especially for the most advanced national economies, to an evolution in time of their export baskets that mimics rather closely that of the globally aggregated basket. In addition, one can expect the aggregated dynamics to show a sort of mean field character compared to the national ones. The relative fluctuations or deviations of individual baskets should be smoothed by aggregation, allowing an easier treatment.

When aggregating yearly exports at the global level, for each product one gets a resultant coinciding with that obtained by aggregating imports. This is not the case when considering the dynamics at the level of single nations and, in this case, one could in principle choose to consider either import or export baskets. The choice of exports is, of course, appropriate if, for example, the aim is to investigate the productive potential of the countries in accordance with general ideas of the economic complexity approach [9]. Here, more consistently with the previous investigation of aggregated exports, we consider, for each country, the basket reporting the sum of yearly exports and imports for each product. This choice is consistent with the aim of describing the growth of the economies, which is related to the increments of both imports and exports.

The purpose of the present work is to describe some relevant features revealed by the dynamic treatment of such composite baskets for the most developed countries, namely those of the G7. The aspects on which we want to focus are related to the structure of the calibrated evolution models, and to their solutions in suitable conditions. A basic feature of the models is that they embody, as an essential element, interaction effects between products. These interactions correspond to transfers of resources between products and allow representations in terms of directed, weighted networks connecting these products. The meaning of these networks is strictly dynamical. This makes them completely different from previously considered networks relating products in the economic complexity approach. We describe here in detail the structure of these networks and discuss what this structure can teach us, especially with reference to the centrality of the various products.

The transfers of resources between different products individually experiencing time-correlated variable market conditions lead also to an autonomous, specific contribution to the average growth [10]. This effect is known for example in portfolio optimization [11], where one tries to increase growth by shifts of investments aimed at profiting at each time of the assets offering highest return. By simulations of our model, we are able to perform interesting counterfactual analyses aimed at establishing up to what extent a variation in the rate of transfers with respect to the historically calibrated one could have changed the growth performance of a given country. This type of analysis can give useful indications about the growth potential of a country.

## 2. The Model

Data for yearly exports and imports were taken from the international trade data furnished by the National Bureau of Economic Research [12] and cover a period of 39 years from 1962 to 2000. The products are classified on the basis of the Standardized International Trade Code at 3-digit level (SITC-3) and trades are reported in US-dollars. We limit here our consideration to the countries of G7 (Canada, France, Germany, Italy, Japan, the United Kingdom, and the USA) and, as a benchmark, we consider the global aggregate exports realized by all countries worldwide (note that imports and exports for each one of these countries have been recorded as imports from and export towards the rest of the world). We denote as $Z_{p,n}^{c}$ the total value (in thousands of current US-dollars) of the product category *p* ($p=1,2,\ldots ,M^{c}$) traded (import plus export) in the year *n* (n=0,1,…,T with T=38) by country *c*. The number of products, $M^{c}$, varies from country to country and is reported in Table 1. When referring to worldwide aggregated data, the superscript *c* is implicitly assumed to mean world.

**Figure 1:**
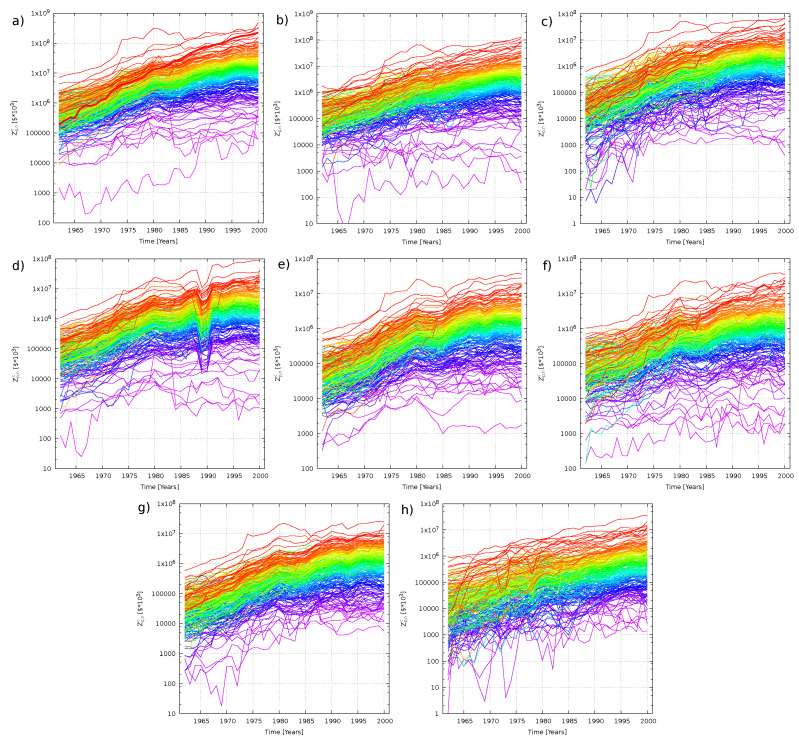
Time evolution of the trades $Z_{p,n}^{c}$ of the World (**a**); the USA (**b**); Japan (**c**); Germany (**d**); France (**e**); the UK (**f**); Italy (**g**); and Canada (**h**). The wavelengths of the colors used are directly proportional to $z_{p}^{c}$.

As already noticed in previous papers [7,8], a key feature of the organization of economies consist in the fact that the various products, besides showing an approximate average exponential growth, have a rather stable ranking over the whole considered period. In fact, if we assign to each product *p* of a given country a color, the wavelength of which is proportional to the fraction of the value of product *p* over the whole country basket, $\Omega_{n}^{c}\equiv \sum_{p=1}^{M^{c}}Z_{p,n}^{c}$, averaged over the last 10 years covered by the database,

(1) $$z_{p}^{c}\equiv \frac{1}{10}\sum_{n=29}^{38}\frac{Z_{p,n}^{c}}{\Omega_{n}^{c}}\;,$$

a rainbow image is perceived when plotting the exports as a function of time (see Figure 1).

The interpolations shown in Figure 1 resemble those of geometric Brownian motions: On average they show similar drifts and fluctuations keeping amplitude constant on a logarithmic scale. This suggests to define average growth as [10]:

(2) $$\lambda_{T}^{c}=\frac{1}{T\cdot M^{c}}\sum_{p=1}^{M^{c}}\log\left[\frac{Z_{p,T}^{c}}{Z_{p,0}^{c}}\right]$$

In Table 1 (second column), we report the estimated growth for the world and for each country. As we can see, Canada and Japan are the countries that experienced the largest average growth, while the United Kingdom is the country that grew the least among the G7.

Since yearly trade records result from variations in much shorter periods, we switch to continuous time *t* in year units (with t=0 corresponding to 1962): The trade values in the year preceding time *t* is now indicated with $Z_{p}^{c}\left(t\right)$. Thus, $Z_{p,n}^{c}$ gives a discrete representation of $Z_{p}^{c}\left(t\right)$ with a point for every year in the database. Taking inspiration from a former paper of Goudré et al. [10] and following Ref. [7] the minimal model describing how the values of various trades evolve in time can be written as a set of Stochastic Differential Equations (SDE):

(3) $$\partial_{t}Z_{p}^{c}\left(t\right)=\left[\eta_{p}^{c}\left(t\right)+\mu^{c}\left(t\right)\right]Z_{p}^{c}\left(t\right)+\sum_{p^{\prime }\neq p}\left[J_{pp^{\prime }}^{c}Z_{p^{\prime }}^{c}\left(t\right)-J_{p^{\prime }p}^{c}Z_{p}^{c}\left(t\right)\right]\;,$$

where $\mu^{c}\left(t\right)$ represents a deterministic drift, accounting for the average growth of the exports (including the inflationary one), and $\eta_{p}^{c}\left(t\right)$ is a multiplicative noise representing the variability of conditions faced by different products at different times. Such variable conditions may depend on many complex causes which can be both “internal” to the country itself and “external”, i.e., due to dynamics in countries with which goods are traded. Finally, the coupling terms $J_{pp^{\prime }}^{c}$ describe the shift of resources from production *j* to production *i*.

Stochastic differential equations similar to Equation (3) were used to describe other problems like population and evolutionary dynamics [13,14], portfolio strategies [11], interface growth [15], or optimal pinning of vortices by random defects in materials [16,17]. An interesting aspect of Equation (3) is the fact that, if the noise $\eta_{p}^{c}\left(t\right)$ is correlated in time, as it is natural to expect, its combination with the coupling terms proportional to $J_{pp^{\prime }}^{c}$ may induce a nonzero average growth, even in the absence of deterministic trends driving the single productions [10].

The deterministic drift, $\mu^{c}\left(t\right)$, represents the average growth of the import plus exports in a given country in the absence of contributions generated by the interplay between the correlated noise and transfer terms. The time dependency comes from the need to include inflationary effects due to the fact that import-export values are expressed in the current currency of the specific year. Thus, we write:

(4) $$\mu^{c}\left(t\right)={\bar{\mu }}^{\;c}+I\left(t\right)\;,$$

in order to separate the real average contribution to the drift, ${\bar{\mu }}^{\;c}$, from the inflationary one, I(t), which can be read as a yearly step-wise function, the values of which are taken from the Organization for Economic Cooperation and Development (OECD) [18] and reported in Table 2.

To take into account the variability of conditions to which the production and acquisition of a given product are subjected, Equation (3) contains a multiplicative noise, $\eta_{p}^{c}$, that, together with the deterministic drift, lets the quantities $Z_{p}^{c}\left(t\right)$ perform a sort of geometric Brownian motion. As we mention, this noise has to be correlated in time and our choice, similar to what has been done in Ref. [10], is for an exponential correlation with a characteristic time $\tau^{c}$ which represents the typical duration of opportunity/crisis periods. Since the various products are reasonably clustered in sets facing similar external conditions, we also introduce a correlation between products. Thus, the noise has zero average, $\langle \eta_{p}^{c}\left(t\right)\rangle =0$, and its correlator reads:

(5) $$\langle \eta_{p}^{c}\left(t_{1}\right)\;\eta_{p^{\prime }}^{c}\left(t_{2}\right)\rangle =c_{pp^{\prime }}^{c}\;\frac{{\left(\sigma^{c}\right)}^{2}}{\tau^{c}}\;e^{-|t_{1}-t_{2}|/\tau^{c}}\;,$$

where $\sigma^{c}$ weights the importance of the stochastic part of the dynamics, and $c_{pp^{\prime }}^{c}$ are the elements of the correlation matrix constructed from the variations at equal times of the different exports in the database [7] (see Section 5 for further details).

Finally, we put the coupling terms equal to:

(6) $$J_{pp^{\prime }}^{c}=G^{c}z_{p}^{c}\left|c_{pp^{\prime }}^{c}\right|\;.$$

where $c_{pp^{\prime }}^{c}$ are again the elements of the correlation matrix entering in the noise, and $G^{c}$ is a coupling constant that regulates the magnitude of the transfer of resources among different products. By considering the factor $|c_{pp^{\prime }}^{c}|$ as a proxy for inverse distance, Equation (6) is consistent with the gravity law, often used for estimating transfer rates in economics [20,21]. The proportionality of $J_{pp^{\prime }}^{c}$ to $z_{p}^{c}$ guarantees that, for t→∞, $Z_{p}^{c}\left(t\right)∼z_{p}^{c}$.

**Figure 2:**
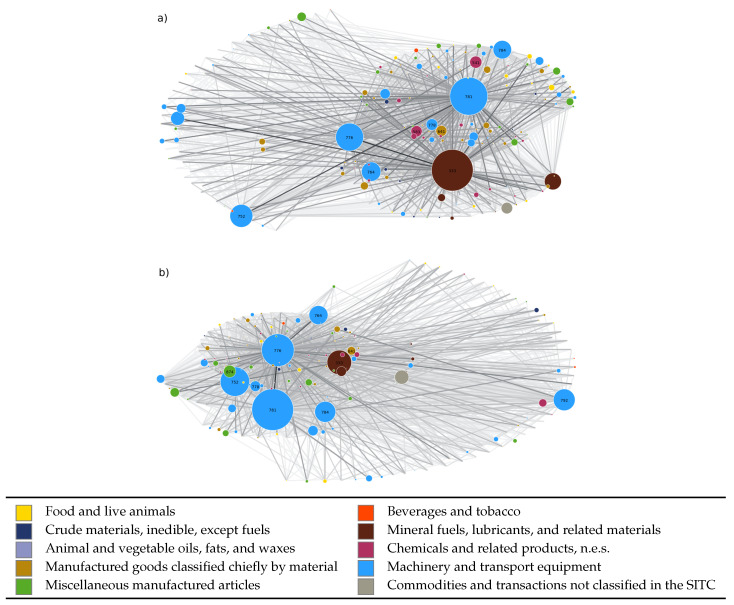
Representation of undirected graphs $U^{WOR}$ (panel (**a**)) and $U^{USA}$ (panel (**b**)) associated with the corresponding ${\tilde{J}}_{pp^{\prime }}^{c}=\max\left\{J_{pp^{\prime }}^{c},J_{p^{\prime }p}^{c}\right\}$ matrices. Since $U^{c}$ are fully connected graphs with approximately $M^{c}\left(M^{c}-1\right)∼5\times 10^{4}$ edges, here we reported only the 10% of the strongest links, which in turn are colored with a palette that is lighter for the weakest among these. The size of the nodes is directly proportional to the value of the ranking $z_{p}^{c}$ associated with the product *p* (the SITC code is highlighted for the most central nodes, see Table 3), while the colors are representative of the macro-category of products illustrated in the legend.

The network specified by the $J_{pp^{\prime }}^{c}$’s is deeply different from other networks proposed in the economic complexity literature as that based on products similarities [9]. In the latter, for instance, *apples* and *pears* are strongly connected because they need the same infrastructures and undergo similar production processes. Such kinds of similarities are indirectly present in our case as a secondary effect inherent to the correlation matrix, which in turn also takes into account the fact that a fluctuation in the trade of *oil* at a certain time is likely going to affect the production of *apples*, *pears*, and many other products. However, an even more important feature of the matrix of transfer rates is the proportionality $J_{pp^{\prime }}^{c}\propto z_{p}^{c}$, which strongly weights the influence of each single product on the global dynamics. Indeed, if a given product $p^{\prime }$ experiences favorable conditions for growth, in force of this proportionality, part of its extra gain tends to be mostly redistributed towards nodes with larger $z_{p}^{c}$. A proper estimate of how effective this mechanism is, requires to also take into account proper notions of centrality of the directed network. The next section is partly devoted to such discussion. At the same time, given the proportionality of $J_{pp^{\prime }}^{c}$ to $z_{p}^{c}$, the structure of our network is such that the most traded products are also the most central nodes (see Figure 2).

## 3. Results

### 3.1. Matrix of Transfer Rates

For each country and for the World, the directed network having products as nodes and links is quite complex. Figure 2 gives a partial and undirected visual representation of the network structure in the case of the USA and the world. The links, ${\tilde{J}}_{pp^{\prime }}^{c}$, of the undirected graphs, $U^{c}$ and $U^{WOR}$, are built with a maximum criterion: ${\tilde{J}}_{pp^{\prime }}^{c}=\max\left\{J_{pp^{\prime }}^{c},J_{p^{\prime }p}^{c}\right\}$. At a first glance, the qualitative structure of the two networks appears very similar: There are few nodes (about 2% of the total number) that are central (degreewise) in the graphical representation, with the remaining nodes being connected almost exclusively to the more central ones in a fashion similar to scale-free networks [22,23]. In both cases the central nodes are given by the same categories of goods, namely oil, cars, machinery, and electronics related products. Finally, the USA seem to have an economy thoroughly dominated by machinery and electronics, and, quite surprisingly, the oil related nodes are substantially smaller than in the case of the world network.

A more quantitative analysis to highlight these analogies and differences can be performed by determining a suitable node centrality measure. The $J_{pp^{\prime }}^{c}$ matrix has strictly positive real entries, that can be considered as the weights of the links of a full directed graph. To properly exploit such a feature, we chose to make use of the Kleinberg’s authority score [24]. In Table 3, we show the top 10 commodities with respect to Kleinberg’s authority: As we can see, 8 out of 10 products are present in both charts and only swap position in ranks, confirming that the two networks are very similar to each other. It is interesting to notice that the ranking of products in terms of authority score does not differ very much from that in terms of $z_{p}^{c}$. So, the structure of our network is such that the most traded products are also the most central nodes (see Figure 2). Interestingly, Sharma et al. have recently shown that a very similar structure arises in the financial network at sectoral level by using a methodology based on multi-layered networks [25,26]: In fact, their results show that there exists a one-to-one mapping between the economic size of the sectors and their centrality in the corresponding financial network.

**Figure 3:**
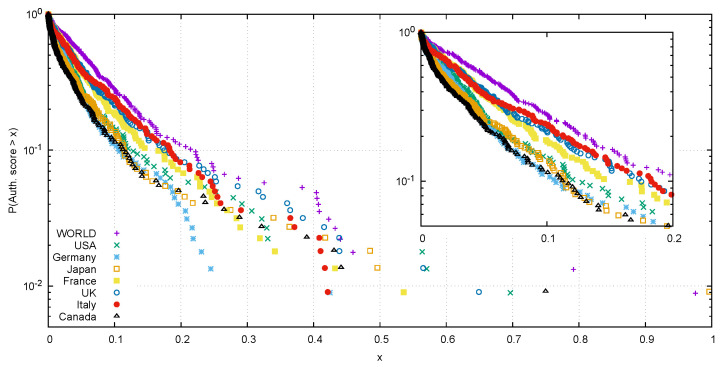
Empirical survival distribution function (ESDF) of the products’ authority evaluated over the graphs associated with the $J_{pp^{\prime }}^{c}$ network of the world and the countries member of the G7. For the majority of the less central products the trend follows an exponentially decaying law, with all the countries showing a curve slightly steeper than the one of the world. Top ranked products of the world and the USA are reported in Table 3.

In Figure 3, we plotted the empirical survival distribution function (ESDF) of the authority scores for the networks of the World and of all the G7 countries. One notices that the world’s network has two nodes with a very high value (781 and 333), while the USA have only one (776). Another interesting feature pointed out by Figure 3 is the initial exponential decay of all the authority scores ESDF. This is another property that our product networks have in common with scale-free networks. All the networks of the G7 countries present a distribution steeper than the world’s one. Taking inspiration from work related to the vulnerability of networks [27], steepness can be interpreted as an alternative instability indicator [28], since concentrating high values of centrality in few products will result in an exposure of the country to major risks in the eventuality of a crisis striking such sectors (negative trends will spread very easily to the rest of the nodes). These scenarios could be in principle tested with our dynamic model, through simulations of hypothetical setbacks of high-centrality products.

### 3.2. Calibrated Parameters

For every G7 country, we performed a calibration procedure in order to determine the values of the model parameters that best fit the historical data. In Table 1, we show such values with the associated errors. We note that $\sigma^{c}$ assumes values in the interval $\left[0.1,0.2\right]\;y^{-1/2}$, with Canada and Japan showing the highest ones, and the world the smallest. The latter feature has to be expected since multiplicative noise should get reduced by the aggregation process. The values of the parameter $G^{c}$ of all the countries have comparable magnitudes. In the next section we will investigate more accurately the role played by such parameter in determining the overall average growth, $\lambda_{T}^{c}$, of every country.

All the values of ${\bar{\mu }}^{c}$ are of comparable magnitude and are substantially lower than the contribution, which can be ascribed to average inflation. This is approximately $0.08\;y^{-1}$ (see Table 2 in the Section 5). Quite interesting is the case of the UK, the only country showing a negative value for the deterministic drift ${\bar{\mu }}^{c}$: This is probably due to the fact that the UK, in the second half of the twentieth century, did not manage to fully exploit the relationship with its trade partners as well as the other countries did. Nevertheless, its inner trade network is still good because it shows an average growth similar to that of the other countries.

### 3.3. Counterfactual Analysis and Optimization

Our model allows to distinguish three distinct contributions to the overall growth. In fact, if we integrate Equation (3) and average over the products we obtain:

(7) $$\lambda_{T}^{c}=h_{T}^{c}\left(G^{c}\right)+{\bar{\mu }}^{c}+\frac{1}{T}\int_{0}^{T}I\left(t\right)\;dt$$

On top of the two deterministic contributions due to ${\bar{\mu }}^{c}$ and the average inflation, we have the term $h_{T}^{c}\left(G^{c}\right)$. This results from the integration of the transfer terms and can be estimated from historical data by discrete summations. Its magnitude depends sensibly on $G^{c}$, and on the interplay that the resource transfers have with the fluctuations determined by multiplicative noise. Indeed, favorable stochastic fluctuations at a local level, if properly exploited, can spread globally to the rest of the network more efficiently than unfavorable ones. In portfolio optimization, this fact leads to the explore-exploit dilemma [10] of deciding whether to exploit a local opportunity (of amplitude $\sigma^{c}$ and expected duration $\tau^{c}$), or to move towards possibilities offered by other nodes in the network by transferring a percentage of the local investment (at a transfer rate speed controlled by $G^{c}$).

**Figure 4:**
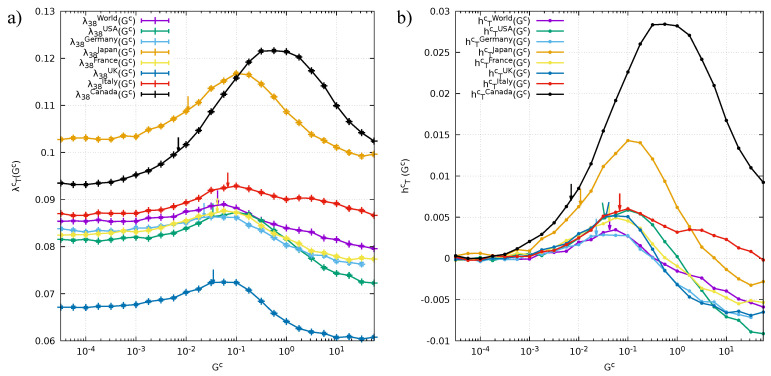
(**a**) Study of the overall growth $\lambda_{T}$ dependence on the parameter $G^{c}$. Arrows indicate, for every country (world), the coordinates of the real historical data; (**b**) Same curves of the previous panel deprived of the deterministic drifts (average inflation and ${\bar{\mu }}^{c}$), hence showing the contribution to the growth $h_{T}^{c}$ given by the cooperative effects of the dynamic network.

It is therefore interesting to study and quantify what the overall growth of the network would have been if we varied the coupling constant of the transfers rate $G^{c}$. Such *counterfactual analysis* produces the plots reported in Figure 4: In panel a we show, for every G7 country and the world, the dependence of $\lambda_{T}^{c}$ on $G^{c}$ (in logarithmic scale). These results are obtained by simulating many times the evolution of every network at fixed, historically calibrated values of ${\bar{\mu }}^{c}$, $\sigma^{c}$ and $\tau^{c}$, and varying only $G^{c}$. For extremely low values of $G^{c}$ the growth is exclusively determined given by the deterministic drifts, and Equation (3) reads:

(8) $$\frac{\partial Z_{p}^{c}}{\partial t}=\left(\eta_{p}^{c}\left(t\right)+\bar{\mu }+I\left(t\right)\right)Z_{p}^{c}\left(t\right).$$

Once integrated in the interval [0,T] and averaged over the products, this equation yields the relation $\lambda_{T}^{c}={\bar{\mu }}^{c}+\frac{1}{T}\int_{0}^{T}I\left(t\right)\;dt$. For increasing values of $G^{c}$, we see that every country produces the same kind of curve: $\lambda_{T}^{c}$ rises until it reaches a peak for some specific value of $G^{c}$, and then starts to fall for large values. Almost every country (the exception is Canada) has a curve that for extremely large values of $G^{c}$ goes beneath the plateau defined by the deterministic drift: This means that in conditions of frenetic transfers the contribution to the growth $h_{T}^{c}$ is negative.

The arrows in the plots indicate the coordinates of $G^{c}$ and $\lambda_{T}^{c}$ determined from the historical data (values shown in Table 1). In order to better compare the intrinsic growth of the network, in Figure 4b we plot the same curves deprived of their drift contributions.

Clearly, every network is characterized by different peak amplitudes, and by different locations of the peaks with respect to the historically calibrated $G^{c}$. For two countries (namely Canada and Japan), the plots indicate a much higher, unexpressed growth potential compared to that of the other countries. Indeed, an even mild increase of $G^{c}$ for them would have produced substantial extra growth. These two countries are also those with the highest historical $h_{T}^{c}$, and this is in agreement with the fact that Japan and Canada have been two emerging economies in the second half of the twentieth century that reached a well established position nowadays (indeed they became G7 members). Looking at the rainbow plots in Figure 1, we also see that for these countries numerous products were out of rank in 1962 and only through the later evolution reached a presumably more stable position in the year 2000. This is in agreement with results obtained for the calibration of the parameter $\sigma^{c}$: As we can see in Table 1 and already remarked above, Canada and Japan are the countries with the two highest $\sigma^{c}$ among those observed. Having a great amplitude of fluctuations, together with a good organization of the transfer rates, can lead to a very high overall growth.

For the other countries, we find that the peak has an amplitude of magnitude comparable to that of the world (just slightly bigger). We also see that the values of the calibrated parameter $G^{c}$ are pretty close to that of the world, while those of Canada and Japan are almost one order of magnitude smaller. Moreover, we find that the value of the historically calibrated $G^{c}$ is rather close to the location of the maximum (as for the world). All these hints tell us that these other countries have an economy which is much more similar to that of the world, because they have been well established since the beginning of the analysis in 1962, while Canada and Japan, as previously stated, underwent big radical changes in this 39 years period that led them to become leading economies in the world scenario.

Finally, we observe that a common feature shared by all countries is that the corresponding calibrated value of $G^{c}$ is always on the left side of the peak. This is an indication of a conservative character of their economies, in the sense that these countries prefer the safety of exploiting the resources rather than exploring new directions of investment through transfers.

## 4. Conclusions

A dynamic description of the evolution in time of the bipartite network connecting countries to the traded goods can be a key to identify interesting complexity features of the economies and of the products. The choice of undertaking such modelization amounts to pushing further some basic points of view of the economic complexity approach [1,2]: Besides assuming that export/import panels should be sufficient to take into account intangible factors of the economies, it is postulated that specifying the composition of the trade baskets should be fully sufficient to account for their time evolution up to the information contained in the deterministic drift and the noise. The dynamic insight one can gain is independent from, and complementary to, that provided by analyses of the Fitness complexity approach [3,4,5,6]. Here we tried to give an account of these features and of the perspectives they are opening, by considering the application of our stochastic differential system of equations to the case of the G7 countries and to the data aggregated for the whole world. The relevant emerging aspects are related to the novel network structure underlying the dynamics of transfers between different productions and to the possibility of performing synthetic simulations and counterfactual analyses. The preliminary results shown should provide a clear indication of the information one can expect to extract by a careful analysis of the networks and of the model dynamics.

## 5. Materials and Methods

### 5.1. Correlation Matrix

The first step in building the correlation matrix is to evaluate the yearly logarithmic returns:

(9) $$R_{p,n}^{c}=\log\left[\frac{Z_{p,n}^{c}}{Z_{p,n-1}^{c}}\right]\;,$$

which we standardize exploiting the data of all the available years:

(10) $$r_{p,n}^{c}=\frac{R_{p,n}^{c}-{\langle R_{p,n}^{c}\rangle }_{n}}{\sqrt{\mathrm{Var}{\left[R_{p,n}^{c}\right]}_{n}}}\;.$$

The correlation between the products *p* and $p^{\prime }$ is then defined by:

(11) $$c_{pp^{\prime }}^{c}=\frac{1}{T}\sum_{n=1}^{T}r_{p,n}^{c}r_{p^{\prime },n}^{c}\;.$$

### 5.2. Calibration

In this section, we provide details regarding the calibration procedure that allowed us to find the values of the parameters (shown in Table 1) that best fit the historical data. To the purpose of better readability, here we will drop the $\cdot^{c}$ superscript: Each quantity will be intended as country specific.

The calibration procedure follows mainly the one presented in Ref. [7], with the only difference lying in the approach to estimate the noise parameters σ and τ. The first parameter we are going to calibrate is *G*. Dividing Equation (3) by $Z_{p}\left(t\right)$ and integrating in the time interval $\left[n_{1},n_{2}\right]$ ($n_{1}$ and $n_{2}$ integers), we obtain:

(12) $$f_{p}\left(n_{1},n_{2}\right)=Gg_{p}\left(n_{1},n_{2}\right)+\bar{\mu }+\frac{1}{n_{2}-n_{1}}\int_{n_{1}}^{n_{2}}\eta_{p}\left(t\right)\;dt$$

where we introduced the functions

(13) $$f_{p}\left(n_{1},n_{2}\right)=\frac{1}{n_{2}-n_{1}}\left(\log\frac{Z_{p,n_{1}}}{Z_{p,n_{2}}}+\sum_{n=n_{1}}^{n_{2}}I_{n}\right)$$

(14) $$g_{p}\left(n_{1},n_{2}\right)=\frac{1}{n_{2}-n_{1}}\sum_{\begin{array}{c} n=n_{1}\\ p^{\prime }\neq p \end{array}}^{n_{2}-1}\left[\frac{z_{p}\left|c_{pp^{\prime }}\right|}{2}\left(\frac{Z_{p^{\prime },n}}{Z_{p,n}}+\frac{Z_{p^{\prime },n+1}}{Z_{p,n+1}}\right)-z_{j}\left|c_{pp^{\prime }}\right|\right]$$

Integrals involving $Z_{p}\left(t\right)$ and I(t) are approximated in terms of summations because of the discrete nature of the data at our disposal.

Equation (12) establishes a linear relation between these two functions, therefore we can perform a linear regression of the scatter plot *f* vs. *g* to estimate the value of *G*. As explained in [7], the random source makes the points with coordinate $g_{p}$ close to zero not reliable for the calibration of *G*. By calibration of synthetic histories with known $G^{c}$, we found that considering the 1/10 of the data with highest $|g_{p}|$ value in the regression will provide us with the correct *G* value with a confidence level of the 10%. The intercept obtained with the regression is a first estimate of the drift $\bar{\mu }$, however, since we excluded the points with lower $|g_{p}|$, it is not a very accurate one. After calibrating σ and τ we will be able to perform a more accurate calibration of the drift.

In order to calibrate these two parameters, we first rearrange Equation (12) by moving to the left side the $Gg_{p}$ term, and we evaluate the variance of the resulting equation. Given $n_{2}=n$ and $n_{1}=0$ we obtain:

(15) $$v\left(n\right)=n^{2}\mathrm{Var}\left[f_{i}\left(0,n\right)-Gg_{i}\left(0,n\right)\right]=2\sigma^{2}\left(n+\tau \left(e^{-n/\tau }-1\right)\right)$$

We find the values of the parameters by fitting the empirical variance with the function on the r.h.s. of the equation. Exploiting the fact that the characteristic time is short compared to the time interval of 39 years, we find that the exponential term $e^{-n/\tau }\to_{n\gg \tau }0$. As a consequence, we perform a linear regression analysis of Equation (15), neglecting data for which $e^{-n/\tau }/v\left(n\right)<1\%$. Eventually, we calibrate the remaining parameter $\bar{\mu }$ through repeated synthetic simulations (see below) of the system of equations deprived of the deterministic drift $\bar{\mu }$ itself. We can in fact exploit the growth Equation (7), which in this case reads as $\lambda_{T}^{*}=h_{T}\left(G\right)+\sum_{t=0}^{T}I_{t}$. Thus, one can find the value of $\bar{\mu }$ that reproduces correctly the growth from the historical data by difference:

(16) $$\bar{\mu }=\lambda_{T}-\lambda_{T}^{*}$$

### 5.3. Numerical Integration

Equation (3) is a stochastic differential equation (SDE) characterized by a colored Gaussian noise that evolves according to:

(17) $$\eta_{p}\left(t\right)=\rho \eta_{p}\left(t-\;dt\right)+\sqrt{1-\rho^{2}}\frac{\sigma }{\sqrt{\tau }}\xi_{p}\left(t\right)$$

where $\rho \equiv e^{-\;dt/\tau }$ and $\xi_{p}\left(t\right)$ is a zero-mean Gaussian noise with correlation $\langle \xi_{p}\left(t\right)\xi_{p^{\prime }}\left(t^{\prime }\right)\rangle =c_{pp^{\prime }}\delta \left(t-t^{\prime }\right)$. The Cholesky decomposition (see for instance [29]) of the matrix $C\equiv c_{pp^{\prime }}$ allows us to obtain a noise with such properties. In detail, we perform the $C={\mathrm{LDL}}^{T}$ decomposition, which is ideal in this case because of the relatively large size of the matrices involved (it does not require the evaluation of any square roots in the diagonal terms, which, if excessively small, could become negative because of computing precision issues). This decomposition provides us with a vector of correlated Gaussian noises $\vec{\tilde{\xi }}$ starting from a vector of independent Gaussian noises $\vec{\tilde{\xi }}$:

(18) $$\vec{\xi }={\mathrm{LD}}^{1/2}\vec{\tilde{\xi }}$$

Substituting the correlated noise in Equation (3) and discretizing gives us:

(19) $$\Delta Z_{p,t}=a_{p}\left(t,{\vec{Z}}_{t}\right)\;dt+b_{p}\left(t,{\vec{Z}}_{t}\right)\Delta W_{p,t}$$

where we introduced the Wiener processes $\Delta W_{p,t}=\sqrt{\Delta t}\xi_{p,t}$ and the two coefficients:

(20) $$a_{p}\left(t,{\vec{Z}}_{t}\right)=\left[\sum_{p^{\prime }\neq p}\left(J_{pp^{\prime }}\frac{Z_{p^{\prime },t}}{Z_{p,t}}-J_{p^{\prime }p}\right)+\rho \eta_{p,t-\Delta t}+\bar{\mu }+I_{t}\right]Z_{p,t}$$

(21) $$b_{p}\left(t,{\vec{Z}}_{t}\right)=\sqrt{1-\rho^{2}}\frac{\sigma }{\sqrt{\tau }}\sqrt{\Delta t}Z_{p,t}$$

With the assumption Δt≪τ, different integration prescriptions yield the same results. In all the numerical integrations performed we chose Δt=0.001, which satisfies such a condition. We chose a second order Runge–Kutta scheme for the Itô prescription, suited for systems of equations and characterized by both strong and weak convergence of order 1, which is thoroughly explained in [30].

## Figures and Tables

**Table 1:** Values of the model parameters for the world and each country member of the G7. Errors of the calibration procedure are also reported. Exception is made for $\tau^{c}$, for which the errors obtained from the calibration procedure are not very meaningful since even a great change (up to 80%) in its value influences very little the results and the other calibrated parameters.

	$M^{c}$	$\lambda_{T}^{c}\left[y^{-1}\right]$	$G^{c}\;\left[y^{-1}\right]$	${\bar{\mu }}^{c}\;\left[y^{-1}\right]$	$\sigma^{c}\;\left[y^{-1/2}\right]$	$\tau^{c}\;\left[y\right]$	$h_{T}^{c}\;\left[y^{-1}\right]$
World	226	0.089	0.042±0.001	$\left(6.31\pm 0.05\right)\times 10^{-3}$	0.109±0.002	0.607	$\left(3.46\pm 0.05\right)\times 10^{-3}$
USA	224	0.086	0.034±0.001	$\left(2.25\pm 0.07\right)\times 10^{-3}$	0.153±0.005	2.64	$\left(4.75\pm 0.07\right)\times 10^{-3}$
Japan	220	0.109	0.011±0.001	$\left(2.33\pm 0.01\right)\times 10^{-2}$	0.203±0.009	5.5	$\left(6.43\pm 0.09\right)\times 10^{-3}$
Germany	224	0.086	0.023±0.001	$\left(4.28\pm 0.04\right)\times 10^{-3}$	0.137±0.001	1.67	$\left(2.79\pm 0.04\right)\times 10^{-3}$
France	222	0.087	0.042±0.001	$\left(3.53\pm 0.07\right)\times 10^{-3}$	0.128±0.001	1.70	$\left(4.55\pm 0.06\right)\times 10^{-3}$
UK	221	0.072	0.035±0.001	$\left(-1.192\pm 0.001\right)\times 10^{-2}$	0.18±0.01	12.7	$\left(4.82\pm 0.07\right)\times 10^{-3}$
Italy	221	0.092	0.068±0.002	$\left(7.67\pm 0.09\right)\times 10^{-3}$	0.112±0.004	0.09	$\left(5.88\pm 0.11\right)\times 10^{-3}$
Canada	219	0.100	0.007±0.001	$\left(1.40\pm 0.01\right)\times 10^{-2}$	0.188±0.001	1.02	$\left(7.02\pm 0.10\right)\times 10^{-3}$

**Table 2:** Yearly global inflation rate. Data from 1971 to 2000 are taken from [18], while for the missing initial 9 years period we evaluated a weighted average of the inflation of the 23 countries of highest GDP (taken from [19]). The average value over the 38 years is I=7.92.

Year	$I_{t}$	Year	$I_{t}$	Year	$I_{t}$	Year	$I_{t}$
1963	2.66	1964	2.99	1965	3.28	1966	3.72
1967	3.56	1968	4.11	1969	4.90	1970	5.69
1971	6.92	1972	6.79	1973	9.01	1974	13.85
1975	13.75	1976	11.47	1977	12.15	1978	10.85
1979	12.53	1980	16.70	1981	13.27	1982	11.28
1983	9.25	1984	9.03	1985	8.07	1986	5.30
1987	5.16	1988	6.75	1989	7.64	1990	8.00
1991	8.52	1992	8.04	1993	7.15	1994	8.29
1995	8.46	1996	7.26	1997	6.94	1998	6.83
1999	5.26	2000	5.44	-	-	-	-

**Table 3:** Top 10 products in the world and USA $J_{pp^{\prime }}^{c}$ networks with highest authority score. The value of $z_{p}^{c}$ (defined in (1)) together with its ranking is also reported.

**World**					
**Auth. Rank**	**Auth. Score**	$z_{p}^{c}$ **Rank**	$z_{p}^{c}$	**SITC-3**	**Commodity Description**
1	1	2	0.052	781	Passenger motor vehicles (excluding buses)
2	0.976	1	0.057	333	Crude petroleum and oils obtained from bituminous minerals
3	0.792	3	0.038	776	Thermionic, microcircuits, transistors, valves, etc.
4	0.459	6	0.025	784	Motor vehicle parts and accessories
5	0.440	4	0.031	752	Automatic data processing machines and units thereof
6	0.432	10	0.016	778	Electrical machinery and apparatus
7	0.423	5	0.026	764	Telecommunication equipment, parts, and accessories
8	0.408	13	0.015	641	Paper and paperboard
9	0.407	9	0.016	541	Medicinal and pharmaceutical products
10	0.405	12	0.015	583	Polymerization and copolymerization products
**USA**					
**Auth. Rank**	**Auth. Score**	$z_{p}^{c}$ **Rank**	$z_{p}^{c}$	**SITC-3**	**Commodity Description**
1	1	2	0.054	776	Thermionic, microcircuits, transistors, valves, etc.
2	0.697	4	0.041	333	Crude petroleum and oils obtained from bituminous minerals
3	0.570	3	0.049	752	Automatic data processing machines and units thereof
4	0.564	1	0.069	781	Passenger motor vehicles (excluding buses)
5	0.331	7	0.031	764	Telecommunication equipment, parts, and accessories
6	0.330	6	0.035	784	Motor vehicle parts and accessories
7	0.322	10	0.018	778	Electrical machinery and apparatus
8	0.307	9	0.020	874	Measuring, checking, analysis, controlling instruments, parts
9	0.292	5	0.036	792	Aircraft and associated equipment, and parts thereof
10	0.279	14	0.014	641	Paper and paperboard

## Abbreviations

The following abbreviations are used in this manuscript:

GDP	Gross Domestic Product
GDP_pc_	Gross Domestic Product per capita
CPI	Commodity Price Index (inflation)
SDE	Stochastic Differential Equation
ESDF	Empirical Survival Distribution Function
